# Acute scrotum a dilemma in a 21-month child: a case report

**DOI:** 10.1097/RC9.0000000000000394

**Published:** 2026-03-12

**Authors:** Sudesh Lamsal, Raj Kumar Shrestha, Amol Gurung, Aastha Shrestha, Sabin Poudel, Aashutosh Pokharel

**Affiliations:** a Department of Surgery, Nepalese Army Institute of Health Sciences, Kathmandu, Nepal; b Department of Surgery, Nepal Medical College, Kathmandu, Nepal

**Keywords:** acute, case report, orchidectomy, scrotum, testicular torsion, testis

## Abstract

**Introduction:**

Acute scrotum in young children presents a diagnostic challenge, as clinical assessment is often limited and symptoms may be nonspecific. Although scrotal trauma is uncommon, it can be associated with serious underlying conditions such as testicular fracture or torsion. Early identification and timely surgical intervention are critical to prevent testicular loss.

**Case presentation:**

We report the case of a 21-month-old toddler boy who presented with left scrotal pain and swelling following minor blunt trauma. Ultrasonography revealed a loculated collection, and absent vascularity in the left testis, suggestive of testicular fracture. Due to suspicion of testicular rupture, emergency scrotal exploration was performed. Intraoperatively, the patient was found to have left testicular torsion. The testis was nonviable, and a left orchidectomy was carried out.

**Discussion:**

Traumatic testicular torsion is a rare but important differential diagnosis in pediatric acute scrotum. Ultrasonography is valuable for initial evaluation; however, overlapping features may lead to diagnostic uncertainty. Surgical exploration remains essential when rupture or torsion is suspected.

**Conclusion:**

This case highlights the importance of maintaining a high index of suspicion for testicular torsion in children presenting with acute scrotum after trauma. Prompt evaluation and early surgical intervention are crucial to optimize outcomes.

## Introduction

Scrotal trauma is an uncommon condition, accounting for less than 1% of all trauma-induced injuries. Blunt trauma is the most common cause of scrotal trauma^[^1^]^. Pediatric children are less prone to acute scrotum because they have lower exposure to infective causes of epididymitis, fewer predisposing factors for testicular torsion, and limited trauma exposure compared to adults^[^2^]^. It occurs when sufficient force compresses the scrotal structures against the thigh or pubic bone. Approximately 85% of these injuries include testicular torsion, testicular fracture or rupture, epididymo-orchitis, and torsion of the testicular appendages. Among these, testicular torsion is a rare but critical emergency, with an annual incidence of 3.8 per 100 000 pediatric patients. Acute scrotum is a frequent presentation in emergency settings. Ultrasound is the preferred diagnostic tool for assessing scrotal trauma, as it effectively identifies testicular blood flow, contusions, hematocele, or ruptured tunica^[^3,4^]^. Based on patient presentation and Doppler findings, management may involve conservative measures or surgical exploration. Early surgical intervention is essential to salvage the affected testis and prevent complications^[^4,5^]^.

HIGHLIGHTS

- Acute scrotum in toddlers poses significant diagnostic challenges due to limited history and examination.
- Scrotal trauma can mask underlying testicular torsion, even when ultrasonography suggests testicular fracture.
- Doppler ultrasonography is a valuable initial tool but may not reliably differentiate torsion from traumatic injuries.
- Early surgical exploration is crucial when testicular rupture or torsion is suspected to prevent testicular loss.
- A high index of suspicion for testicular torsion is essential in pediatric acute scrotum following trauma.

Here, we present a case report of testicular fracture with an intraoperative finding of left testicular torsion. This case report has been reported in line with the SCARE checklist^[^6^]^.

## Case

A 21-month-old toddler boy, referred from a primary healthcare center, presented to the emergency department with complaints of pain and swelling in the left scrotum for the past 18 h. The patient had a history of trauma to the perineal region following a fall from a height of 1 foot. The pain was sudden in onset, progressive, and accompanied by swelling in the scrotum. There was no history of hematuria, nausea, vomiting, fever, or any significant past medical history.

**Figure 1.**
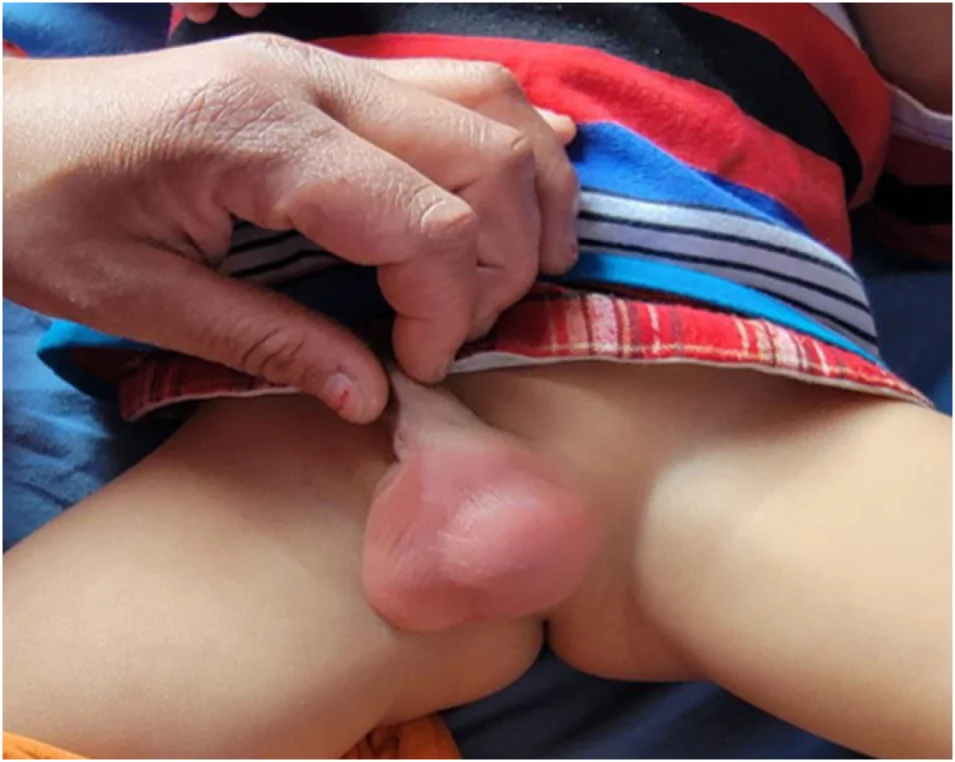
Image showing an edematous and tender left testis, while the right testis appears normal.

**Figure 2.**
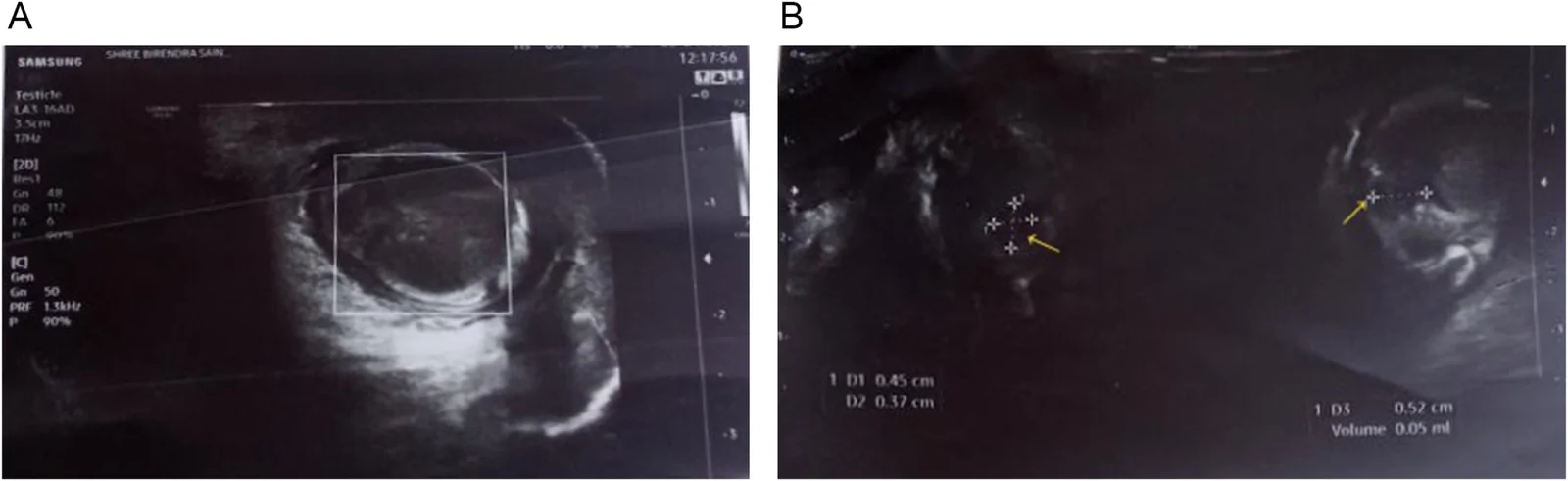
(A, B) Scrotal ultrasound demonstrating a testicular fracture with a loculated collection and no detectable vascularity in the left testis.

On clinical examination, the right testis appeared normal, while the left testis was edematous and tender as shown in Figure 1. The abdominal examination revealed no abnormalities. Scrotal ultrasound was suggestive of a testicular fracture with a loculated collection and no demonstrable vascularity in the left testis (Figure 2).

Emergency scrotal exploration was planned due to a clinical suspicion of testicular rupture. During exploration, testicular torsion on the left side was identified. The left testis was deemed nonviable, necessitating a left orchidectomy. The hematoma was evacuated, and hemostasis was achieved. The tunica vaginalis and dartos muscles were sutured using vicryl, and the skin was closed with catgut sutures.

## Discussion

Testicular trauma is rare in children under 10 years of age, accounting for only 1.5% of cases across all age-groups^[^7^]^. Blunt trauma to the scrotum can result in severe injuries, ranging from testicular hematoma to torsion, fracture, rupture, and avulsion^[^5^]^. In cases of acute scrotum, obtaining a detailed history and performing a clinical examination are often insufficient for diagnosis, as external genitalia can be challenging to assess. This limitation occasionally leads to misdiagnosis^[^5,8^]^.

Ultrasonography is a reliable, effective, and noninvasive imaging technique for diagnosing traumatic scrotal injuries^[^9^]^. Doppler ultrasonography, in particular, has demonstrated a sensitivity of 94% and specificity of 96% for diagnosing torsion, 87% and 89% for hematocele, and 100% and 97% for testicular avulsion, respectively^[^9,10^]^.

Our case involved a 21-month-old toddler boy presenting with acute scrotum. In this case, ultrasonography proved more accurate than a detailed history and clinical examination due to the patient’s young age. The ultrasound revealed a linear hypoechoic band extending across the testicular parenchyma, an intact tunica albuginea, and heterogeneously hypoechoic contents in the left testis. Additional findings included an edematous left testis and a bulky left epididymis with heterogeneous echotexture, suggesting testicular fracture with no demonstrable vascularity.

**Figure 3.**
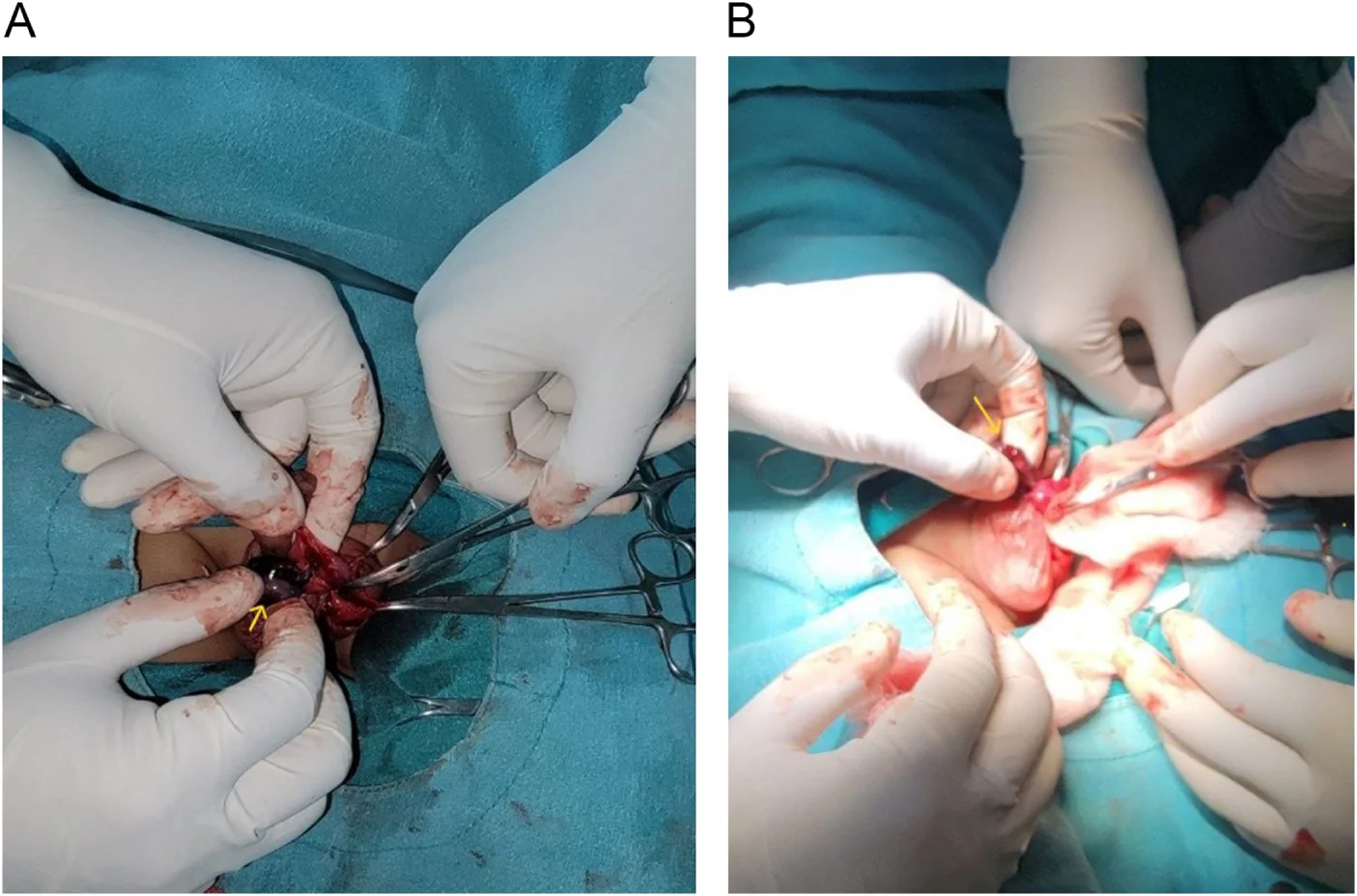
(A, B) Intraoperative findings revealing left testicular torsion.

When testicular rupture is suspected, urgent surgical exploration is necessary to evacuate hematoma, perform tissue debridement, and repair defects, even if Doppler ultrasonography suggests a fracture. Surgical exploration is preferred over conservative management, as the latter is associated with a higher orchidectomy rate of 45%^[^7,11^]^. In this case, surgical exploration was pursued under suspicion of testicular rupture. However, intraoperative findings revealed left testicular torsion, as shown in Figure 3.

The salvage rate of the testis in cases of torsion is significantly higher if exploration occurs within 5 h of trauma. The success rate decreases markedly after 12 h, with some studies indicating that organ salvage is rarely possible beyond this window^[^12^]^. Likewise, due to delayed presentation, the organ could not be salvaged in our patient.

## Conclusion

Testicular torsion is an emergency condition requiring emergency surgery; thus, time is of essence. In resource-poor countries, delayed presentation can lead to the testis being unsalvageable. Diagnosis can be challenging due to an unreliable history and difficulty in examination. However, ultrasound is a useful modality for diagnosis.

## Data Availability

The authors confirm that the data supporting the findings of this study are available within the article [and/or] its supplementary materials.
